# Comparison of antimicrobial efficacy of Acacia catechu mouthrinse and HiOra herbal mouthrinse and their influence on Streptococcus mutans count and Lactobacillus spp. count in children with early childhood caries

**DOI:** 10.3205/dgkh000541

**Published:** 2025-04-28

**Authors:** Madhura Joshi, Shruthi B. Patil

**Affiliations:** 1 Pediatric and Preventive Dentistry, Sri Dharmasthala Manjunatheshwara (SDM) Dental College of Dental Sciences & Hospital, Sattur, Dharwad, Karnataka, India

**Keywords:** early childhood caries, ayurvedic mouthrinse, total amount colony forming units, Streptococcus mutans, Lactobacillus spp.

## Abstract

**Background:**

Early childhood caries (ECC) is a chronic, infectious disease affecting young children. Though several preventive methods/measures are, the awareness about the benefits of a Ayurvedic preparation and its limited side effects is high. Thus, aim of this study was to compare efficacy of Ayurvedic formulations in reduction of the micro-organism causing ECC.

**Method:**

In a double-blind randomized, placebo-controlled trial 60 children between 6 and 71 months age with ECC were divided into group I (n=20, control group), group II (n=20, *Acacia catechu* group) and group III (n=20, HiOra group). Unstimulated saliva was collected pre and after 16^th^ day post mouthrinse. The baseline microbiological colony count was performed for *Streptococcus mutans* and Lactobacillus spp. The results obtained were statistically analysed using non parametric test.

**Results:**

There was significant reduction in the amount of colony-forming units (cfu) between the control and the treatment groups. In the HiOra group the reduction of cfu was tendentially greater (p>0.05) than in the *Acacia catechu* group. In the HiOra group only significant reduction of *Streptococcus (S.) mutans* was seen, whereas in the *Acacia catechu* group significant reduction of *S. mutans* and Lactobacillus spp. was seen.

**Conclusion:**

Reduction in total microorganisms was less significant among the experimental groups. However the reduction in total colony count was greater in HiOra followed by *Acacia catechu* and the control group showed lesser reduction value.

## Zusammenfassung

**Hintergrund:**

Die frühkindliche Karies (FK) ist eine chronische Infektionskrankheit, die vor allem Kleinkinder betrifft. Obwohl mehrere präventive Maßnahmen angewandt werden, sollte der Einfluss ayurvedischer Präparate auf die Reduzierung der Mikroorganismen, die für die Entstehung der FK ätiologisch relevant sind, in der Mundhöhle verglichen werden.

**Methode:**

60 Kinder im Alter von bis zu 71 Monaten mit FK wurden in einem doppelblinden RCT den Gruppen I (n=20, Kontrollgruppe), Gruppe II (n=20, *Acacia catechu*-Gruppe) und Gruppe III (n=20, HiOra-Gruppe) zugeteilt. Unstimulierter Speichel wurde vor der ersten Mundspülung und am 16. Tag nach der Mundspülung gesammelt. Die Ausgangskoloniezahl wurde für *Streptococcus (S.) mutans* und Lactobacillus spp. bestimmt. Die Ergebnisse wurden mit Hilfe eines nichtparametrischen Tests statistisch ausgewertet.

**Ergebnisse:**

Die Gesamtkoloniezahl wurde in beiden Behandlungsgruppen im Vergleich zur Kontrollgruppe signifikant reduziert. Bei Spülung mit HiOra war die Reduktion tendentiell größer (p>0,05) als nach Spülung mit *Acacia catechu*. In der HiOra-Gruppe wurde nur *S. mutans* signifikant reduziert, während in der *Acacia catechu* Gruppe *S. mutans* und die Lactobacillus spp. signifikant reduziert wurden.

**Schlussfolgerung:**

Die Verringerung der Gesamtkoloniezahl unterschied sich zwischen den Behandlungsgruppen nicht signifikant. Allerdings war die Reduktion der Gesamtkoloniezahl durch HiOra größer, gefolgt von *Acacia catechu*, und die Kontrollgruppe zeigte einen geringeren Reduktionswert.

## Introduction

Maintaining healthy teeth and gums is a lifelong commitment. Since compared to other infectious diseases, tooth decay is not self-limiting. There is a greater probability of caries and associated sequelae in both primary and permanent dentition if a child has caries as an infant or a toddler [1]. It influences how their growth, social appearance, masticatory and deglutition functions. Oral health affects children both physically and psychologically [2].

Early childhood caries (ECC) is defined as the presence of one or more decayed (non-cavitated or cavitated lesions), missing or filled (due to caries) surfaces, in any primary tooth of a child aged 71 months or younger [3]. It is a multifactorial disease consequent to the interaction of cariogenic microorganisms, exposure to carbohydrates, inappropriate feeding practices, social and economic factors, neglect of the parents due to career concerns or ignorance, oral hygiene and hampers the overall health and growth of a child and reduces the quality of life [4].

Compared to developed countries where the prevalence rate of EEC is around 1–12% which is in contrast to the developing countries where the rate is expected to be around 44% for 8- to 48-month olds [5], [6], almost at epidemic proportions in the developing countries due to high consumption of simple refined sugars.

ECC is a complex disease of multifactorial origin. The factors include susceptible host, diet containing fermentable carbohydrate, presence of dental plaque, increased number of cariogenic micro-organisms such as *Streptococcus (S.) mutans* and Lactobacillus spp. and time. 

In the recent , the awareness about the benefits of ayurvedic or herbal preparation and its limited side effects is on high. There are various studies showing the maintenance of good oral hygiene using ayurvedic/herbal preparations. Green tea mouthrinse was found to bOK significantly better than chlorhexidine mouth rinse against *S. mutans* [7]. Chlorhexidine digluconate (CHG) and herbal mouthwash (HiOra) showed similar anti plaque activity with latter showing no side effects [8]. Tulsi is as effective as CHG and Listerine in reducing the salivary *S. mutans* levels [9].

Considering the age group and the limited side effects in herbal preparations, this study is hypothesized to compare the efficacy of ayurvedic formulations in reduction of the oral microbial load casing ECC. 

## Materials and methods

### Setting

**Figure 1:**
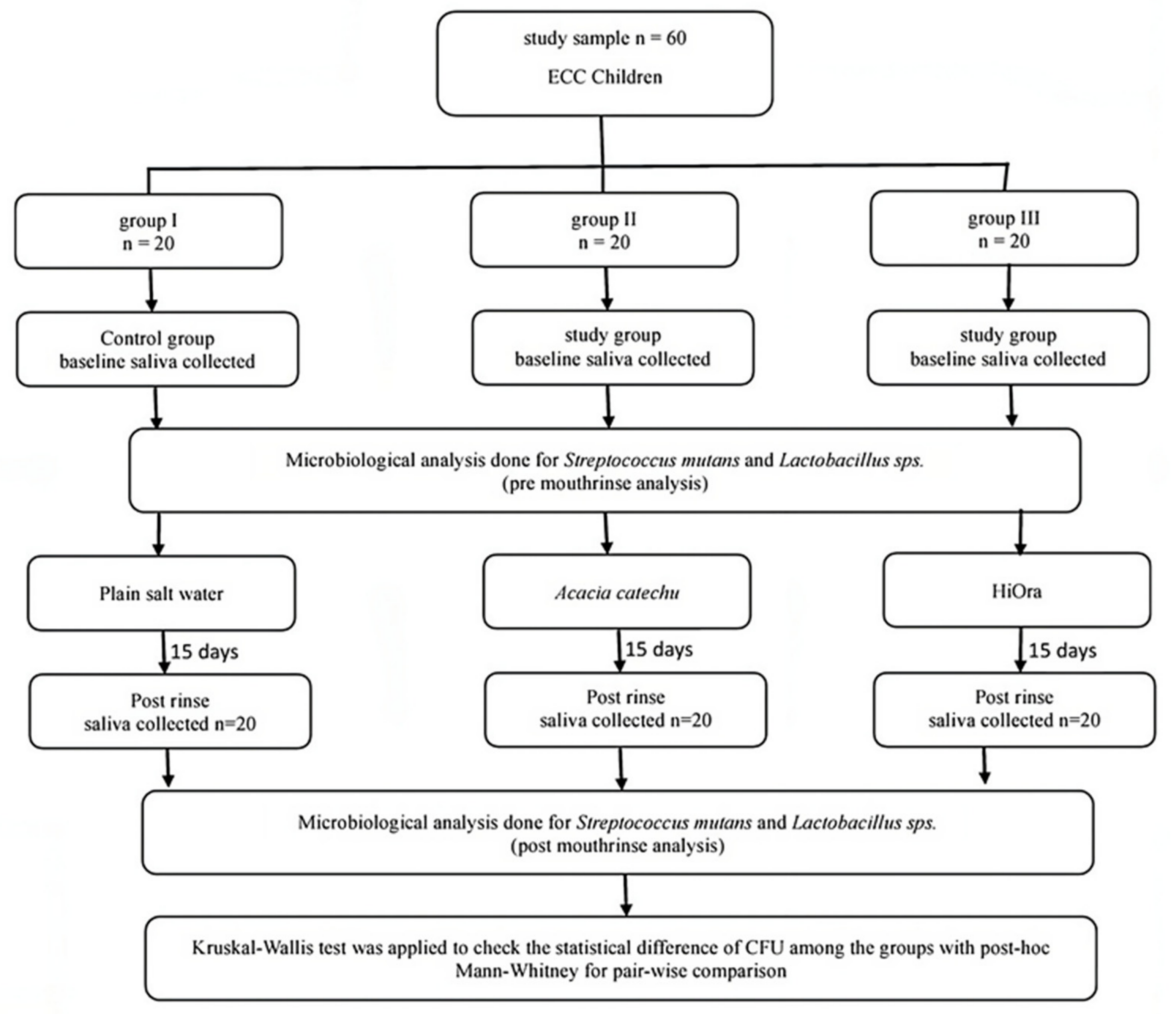
Consort diagram for the randomized trial

The double-blind RCT was performed in the department of paediatric and preventive dentistry, SDM College of Dental Science and Hospital, Dharwad, Karnataka, in collaboration with the department of microbiology research laboratory KLE dental college, Belgaum for microbiological evaluation and with the department of Rasashasthra and Bhaishanjya kalpana, Ayurveda Mahavidyalaya and hospital, Hubli for Ayurvedic formulations. The study was approved by the Institutional Review Board (IRB) of SDM College of Dental Sciences and Hospital, Karnataka, India (IRB NO. 2018/P/PEDO/31). The consort diagram for the randomized trial is given in Figure 1 (Fig. 1).

### Performance of the double-blind RCT

60 children (n=60) who fit the inclusion criteria were randomized into 3 groups with 20 children in each group. Group I, the control, rinse with 10 ml of plain salt water, group II rinse with 10 ml of *Acacia catechu* mouthrinse (Ayurveda Mahavidyalaya, Hubli, India) and group III rinse with 10 ml of HiOra plain herbal mouthrinse (Himalaya^®^ company, Bengaluru, India) 

Inclusion criteria were systemically healthy children entering the out patient department, ECC (more than 4 visible cavitated lesions) and age between 6 to 71 months. Exclusion criteria were medically compromised children, patients who are not willing to participate in the study and white spot lesions or non-cavitated lesions are not considered.

The parent/guardian was explained about the study and an informed consent had been taken prior to the participation. On the day of unstimulated saliva sample collection, the child was instructed to have an early breakfast with a lag of 90 min before the sample collection to avoid any food contamination on the composition of saliva [10].

The children were made to sit in an upright position and 500 µl of unstimulated saliva were collected in sterile containers with the help of sterile swabs. They were run over the teeth surfaces in a standardised direction on both the sides; starting from one surface of swab running from distal surface of lower right primary second molar through the anteriors then to the left lower primary second molars over distal surface. It was then immediately placed into the container with transport media. 

The containers were coded and labelled in accordance to those with mouthrinse bottles, received by each child. The blinded codes were as follows:

1. Group I coded as ‘S’ with serial samples coded as S_1_, S_2_, S_3_, S_4_, S_5_, S_6_ till S_20_.
2. Group II coded as ‘A’ with serial samples as A_1_, A_2_, A_3_, A_4_, A_5_, A_6_ till A_20_.
3. Group III coded as ‘H’ with serial samples H_1_, H_2_, H_3_, H_4_, H_5_, H_6_ till H_20_.

The parents of the children in the groups were provided with coded mouth rinse solution and were advised to dilute 5 ml of respective solution with 5 ml of water. Then with a clean strip of gauge was dipped into the prepared mouth rinse and wiped over all the tooth surfaces; labially/bucally and palatally/lingually for 2 min. This method of usage was advised to be followed twice daily, once in the morning and at night with the interval of 12 hours for 15 days.

Parents were asked to start the procedure from the day after baseline salivary sample was collected. This was to prevent any error caused by mouthrinse that could arise if used on same day of saliva collection. Children neither were restricted from their normal oral hygiene routine nor from the dietary habit. On the 16^th^ day, saliva samples were collected into coded identically with their baseline containers; however an additional alphabet “s” (small letter s) was labelled indicating that the samples were post-mouthrinse samples. The samples were processed in the laboratory on the same day of sample collection to prevent bias. 

### Microbiological evaluation

The baseline cfu counts were performed for *S. mutans* and Lactobacillus spp. with serially diluted pre- and post-mouthrinse samples. They were tested separately using mitis salivarius bacitracin agar and Rogosa agar (Maratha Mandal Microbiological Laboratory, Belgaum, India) enriched with blood agar. These organisms were confirmed by Grams staining and key biochemicals. The cfu were quantified by the same observer under the same conditions and at the same time of the day by a double blinded intra-observer to avoid bias. 

### Statistical analysis

The data was tabulated using Microsoft excel. Kruskal-Wallis test was applied to check the statistical difference of cfu among the groups with post-hoc Mann-Whitney for pair-wise comparison using SPSS (Statistical Package for Social Sciences) version 20 [IBM SPASS statistics (IBM corp. Armonk, NY, USA released 2011)] will be used to perform the statistical analysis. The level of significance was set at 5%. 

## Results

Inter group comparison of amount of cfu was not significant between group I and II (p=0.837), between the Groups I and III (p=1.0), and also between the groups II and III (p=0.978), thus the study showed importance and need to conduct, in order to know the efficacy of each mouthrinse (Table 1 (Tab. 1)).

In the post mouthrinse sample, the amount of cfu showed significant differences between group I and II (p=0.034) and also between group I and III (p=0.034). However, when compared between groups II and III, there was no significant difference (p=0.931). There was significant reduction in the amount of cfu between the control group and the treatment groups p=0.034.

Table 2 (Tab. 2) shows the comparison of cfu between the pre and post mouthrinse sample within the groups. In group I, there was significant reduction in *S. mutans* (p=0.046) and lactobacillus spp. (p=0.00). The amount of cfu showed significant reduction in the postrinse samples (p=0.00).

In group II, there was significant reduction in *S. mutans* (p=0.003) and lactobacillus spp. (p=0.006). The amount of cfu showed significant reduction in the post sample (p=0.000). 

While in group III, the amount of cfu of *S. mutans* was reduced significantly (p=0.14) which was not seen for lactobacillus spp. (p=0.151) and the amount of total cfu showed significant reduction in the post sample (p=0.00). 

## Discussion

Ideal periods for preventing ECC starts at pregnancy and early childhood, as there is evidence that primary tooth decay at young ages are more likely to have an increased caries burden along the continuum of childhood [11]. Effective preventive strategies include reducing maternal levels of *S. mutans*, reducing the vertical transmission of cariogenic microorganisms from mother to infants, early screening of infants and toddlers, promoting regular infant oral hygiene and applying fluoride varnish or other antimicrobial agents [12], [13].

Antimicrobial mouth rinses has become a day routine among all age groups. The awareness about the benefits of ayurvedic preparation in recent era is significantly increasing among the public. This has influenced pediatric dentists to prescribe/use ayurvedic formulations for the age group less than 15 years, especially between 0 to 6 years of age, in the form of topical usage of medicaments like mouth rinse, anaesthetic gel, irrigating solutions for pulpectomy etc. during a time where the conventional mouth rinses are contraindicated due to alcohol content [14].

Beside *S. mutans*, the more prevalent colonization of lactobacilli, the composition of the overall microbiota and several taxa within the oral biofilms of the 3-year-old could be linked to the absence or presence of caries [15]. The combined ‘mutans streptococci + lactobacilli + past caries’ model, with both specificity and sensitivity on 80%, may serve as useful method for selecting children at-risk for targeted intervention [16].

Herbal agents are often considered as safer and are of lower cost than conventional antiseptics [17]. They help in curing gastrointestinal diseases/infections and also have influence on the oral cavity, as this is the main route of administration. 

*Acacia* belongs to the family Leguminosae. It has predominant catechins that include catechin, epicatechin, epicatechin-3-O-gallate, and epigallocatechin-3-O-gallate. Other major secondary products present in the extracts included flavonol glycosides, flavonal dimers, and caffeine [18]. Another ayurvedic preparation was commercially available as herbal mouth rinse HiOra, an extracts of *Terminalia belleric myrobalan* (bibhitaki). It is used in traditional medicine as it has wide spectrum of pharmacological effects associated with biologically active secondary metabolites. It also has *Salvadora persica* (miswak) and oils of *Gaultheria fragrantissima* and *Elettaria cardamomum*. 

The reduction of microbial levels on rinsing with plain water was less significant. This indicates that rinsing alone with plain water did not prove to be beneficial in reducing the microbial load and the use of antimicrobial agents as adjunct to mechanical cleansing is needed for maintaining proper oral health.

The observed decrease in *S. mutans* may be due to *Terminalia belleric myrobalan* in HiOra which is a strong inhibitor of biofilm formation; similar findings were also observed by Yadav et al. [19] with antibiofilm activity against *Streptococcus sobrinus* and *S. mutans*. Though there was decrease in individual *S. mutans*, the decline in the number was not significant among the groups II and III for reduction of *S. mutans* within the given time. This may be due to various extracts of *A. catechu* exhibits antimicrobial activity, these properties are because of catechins and flavonoids present in it. Phytochemical bioactive compounds, tannins, gallic acid, ellagic acid, glycosides, alkaloids, sterols, catechin, phenol, and flavonoids, all of them possess strong antioxidant properties. However these are present in more of *Acacia catechu* than the HiOra. These results of antimicrobial and antioxidant property are similar to those of Govindarajan et al. [20], Baliga [21], Li et al. [22] and Krishnamurthy et al. [23], which have been conducted both in vitro and in vivo.

Decrease in the expression of pro-inflammatory cytokines TNF-α, IL-1β, and IL-6 is also believed to be caused by dual inhibition of cyclooxygenase and lipo-oxygenase enzymes [24].

On comparison with group III, group II showed reduction of Lactobacillus spp. which could be due to presence of epicatechin and epigallocatechin-3-O-gallate which act protective [25]. Similar results were observed by Joshi et al. [26] and Negi et al. [27] who demonstrated methanol extract of *A. catechu* to exhibit antimicrobial properties against *Bacillus subtilis, Staphylococcus aureus, Salmonella typhi, Escherichia coli, Pseudomonas aeruginosa* and *Candida albicans*.

Studies on immunomodulatory shows that daily consumption of *A. catechu* decoction may provide immunity against infections in general [27]. Phytocompounds from herbal agents also act as immunomodulatory agent by activating host defence mechanism and provide an alternative therapy to conventional chemotherapy [28]. In HiOra, this is contributed by the presence of epicatechin and epigallocatechin-3-O-gallate enzymes that effectively reduces the attachment of biofilm over the tooth surface thus inhibiting the colonisation of microbes.

*Areca catechu* has both antimicrobial and antigenotoxic activities which exhibit the highest protective effect against free radicals. Alkaloid could be responsible for inhibiting the microorganism by impairing the enzymes involved in energy production; interfering with the integrity of cell membrane and structural component synthesis, probably this could be the reason to combat the microorganisms in the current study [29].

There were no significant changes between the three groups on total cfu amounts, compared with post mouthrinse sample, where total cfu amounts showed significant difference between group I and II and also between group I and III. The reduction in cfu amount was less significant among the treatment groups, indicating that both act equally against the microorganisms within the given time. Monga et al. [30] on conducting 3 different experiments evaluated, that the *A. catechu* extract was shown to exhibit increased the activities of the antioxidant enzymes that is epicatechin and epigallocatechin-3-O-gallate in a dose-dependent manner and leading to the release superoxide dismutase, catalase, glutathione peroxidase, glutathione transferase and glutathione reductase [31].

A study by Tseng-Crank et al. [32] concluded *A. ca*t*echu* was an anti-inflammatory agent due to its inhibitory effect on the pro-inflammatory genes on IL-1β, IL-6, cyclooxygenase and tumor necrosis factor. The central controlling factor for genes, the nuclear factor kappa B, also was down regulated [30]. *Salvadora persica* (miswak) assessment of this immediate antimicrobial effect was studied by Bhat [33] where he found that it had significant detrimental effect on the dental caries causing microorganisms. Significant reduction in individual *S. mutans* and Lactobacillus spp. and also in total cfu amount was seen when compared between the control and treatment groups. This indicates that both the treatment droups; *Acacia catechu* (group II) and HiOra (group III) act equally over microorganisms reduction. 

Among the mouth rinses used, reduction in total cfu amount was observed as HiOra>*Acacia catechu*>control group. There was difference among the treatment groups but this difference was not significant. Further research has to be undertaken with more number of samples for better inference. 

## Conclusion

The mild reduction in total cfu amount was seen in the treatment groups as they were seen to act equally over *S. mutans* and Lactobacillus spp. HiOra was superior effective compared to *Acacia catechu*. This justifies the recommendation of using ayurvedic formulated mouth rinses in children with younger age group to overcome the allopathic formula drawbacks. 

## Notes

### Authors’ ORCID

- Madhura Joshi: 0000-0003-3342-5470
- Shruthi B. Patil: 0000-0003-1616-3798

### Competing interests

The authors declare that they have no competing interests.

## Figures and Tables

**Table 1:**
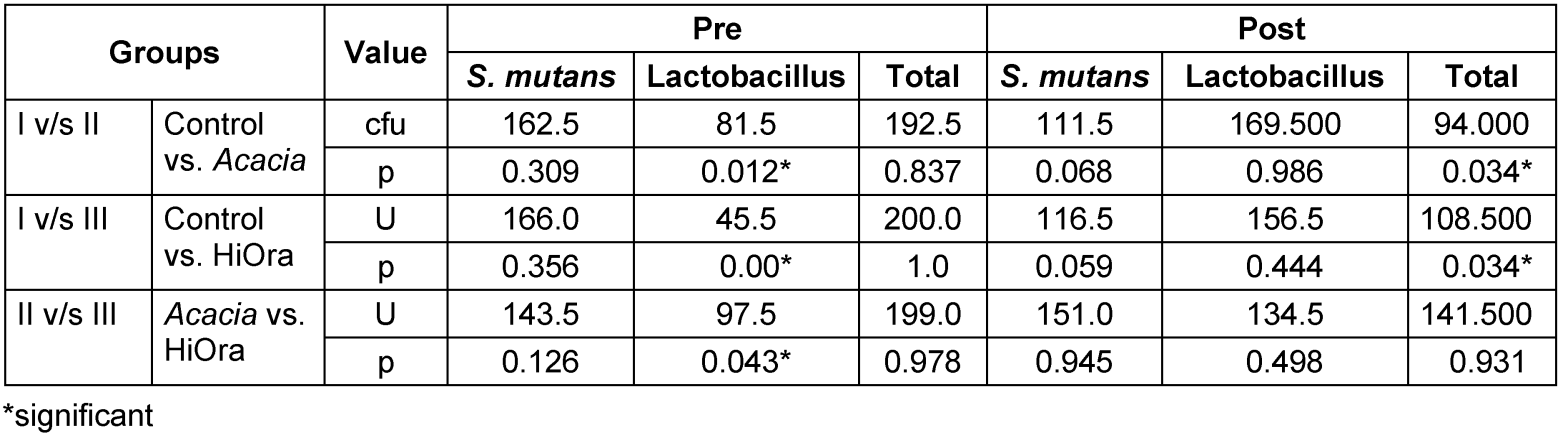
Comparison of cfu between the groups using Mann-Whitney test

**Table 2:**
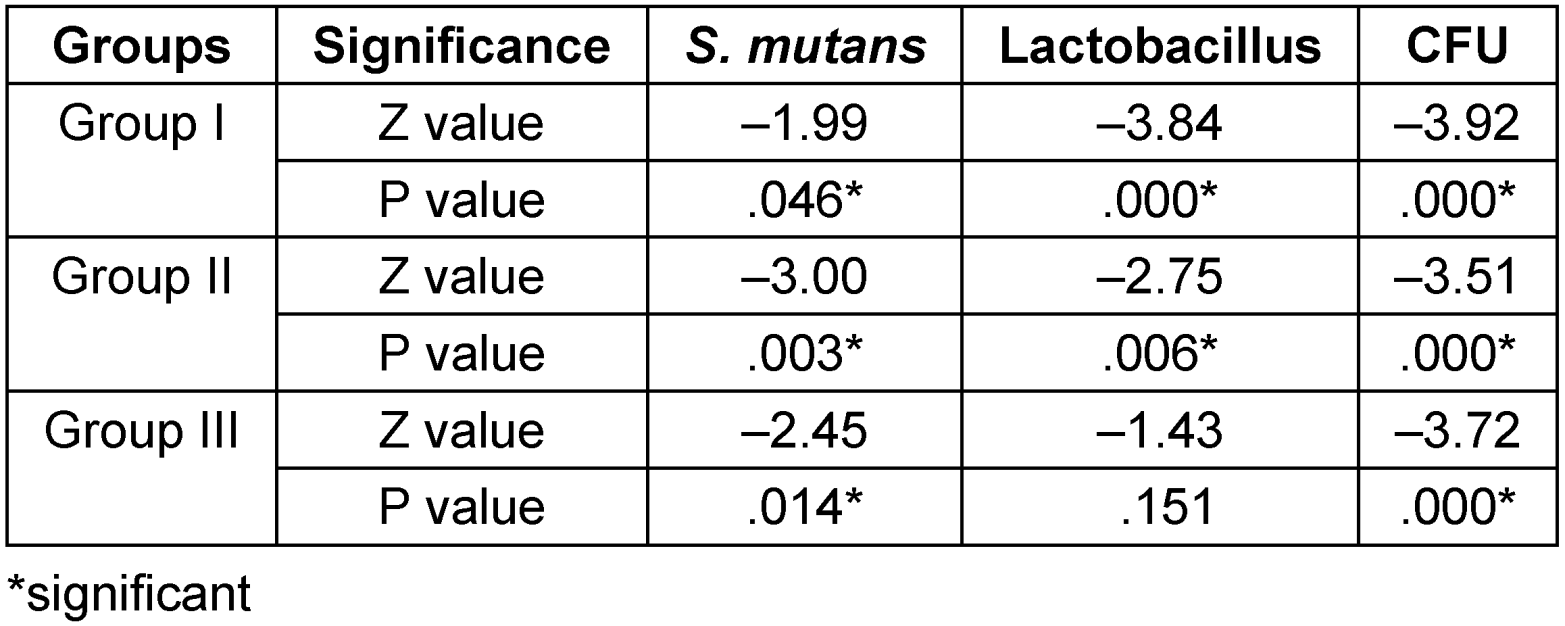
Comparison of pre and post cfu within the group using Wilcoxon sign
